# Gene expression profile induced by arsenic trioxide in chronic lymphocytic leukemia cells reveals a central role for heme oxygenase-1 in apoptosis and regulation of matrix metalloproteinase-9

**DOI:** 10.18632/oncotarget.13091

**Published:** 2016-11-04

**Authors:** Irene Amigo-Jiménez, Elvira Bailón, Noemí Aguilera-Montilla, José A. García-Marco, Angeles García-Pardo

**Affiliations:** ^1^ Cellular and Molecular Medicine Department, Centro de Investigaciones Biológicas, Consejo Superior de Investigaciones Científicas (CSIC), Madrid, Spain; ^2^ Molecular Cytogenetics Unit, Hematology Department, Instituto de Investigación Sanitaria Puerta de Hierro-Majadahonda, Madrid, Spain

**Keywords:** CLL, arsenic trioxide, gene expression profile, HMOX1, MMP-9

## Abstract

CLL remains an incurable disease in spite of the many new compounds being tested. Arsenic trioxide (ATO) induces apoptosis in all CLL cell types and could constitute an efficient therapy. To further explore this, we have studied the gene expression profile induced by ATO in CLL cells. ATO modulated many genes, largely involved in oxidative stress, being *HMOX1* the most upregulated gene, also induced at the protein level. ATO also increased *MMP-9*, as we previously observed, both at the mRNA and protein level. Using specific inhibitors, qPCR analyses, and gene silencing approaches we demonstrate that upregulation of MMP-9 by ATO involved activation of the p38 MAPK/AP-1 signaling pathway. Moreover, gene silencing *HMOX1* or inhibiting HMOX1 activity enhanced p38 MAPK phosphorylation and c-jun expression/activation, resulting in transcriptional upregulation of MMP-9. Overexpression of HMOX1 or enhancement of its activity, had the opposite effect. Cell viability analyses upon modulation of HMOX1 expression or activity demonstrated that HMOX1 had a pro-apoptotic role and enhanced the cytotoxic effect of ATO in CLL cells. We have therefore identified a new mechanism in which HMOX1 plays a central role in the response of CLL cells to ATO and in the regulation of the anti-apoptotic protein MMP-9. Thus, HMOX1 arises as a new therapeutic target in CLL and the combination of HMOX1 modulators with ATO may constitute an efficient therapeutic strategy in CLL.

## INTRODUCTION

Chronic lymphocytic leukemia (CLL) is characterized by the accumulation of malignant CD5^+^ B lymphocytes in the peripheral blood and lymphoid tissues [1, 2]. CLL is very heterogeneous and patients carrying certain bad prognostic markers (del17p13, unmutated IgH_v_) do not respond well to conventional therapies [1–3]. The recent development of more specific agents, such as ibrutinib and idelalisib, has greatly improved the response of most CLL patients [4, 5]. However, the long-term efficacy of these treatments, particularly in refractory CLL cases, is not known and CLL remains an incurable disease. Therefore, it is still crucial to continue searching for new compounds for CLL treatment, especially in the advanced setting.

Arsenic trioxide (ATO) is an efficient therapy in acute promyelocytic leukemia [6] and has shown promising results in other malignancies [7]. We and others have shown that ATO induces apoptosis in all CLL cases, including those with unfavorable prognosis [8–10]. As part of the apoptotic mechanism, ATO induces JNK activation, reactive oxygen species (ROS) generation and PI3K/Akt/NF-κB downregulation in CLL cells [10]. Additionally, we have shown that ATO upregulates membrane-bound matrix metalloproteinase-9 (MMP-9), and that MMP-9 protects CLL cells against the cytotoxic effect of ATO, contributing to the anti-apoptotic effect of stroma [11, 12]. In spite of these advances, ATO functions through multiple mechanisms and the complete response elicited by this agent in CLL cells has not been established.

It is well known that the pro-apoptotic effects of ATO are mainly via generation of ROS and oxidative stress [13]. Heme oxygenase-1 (HMOX1, HO-1) is the main antioxidant enzymatic system in the cell, with crucial roles in the removal of intracellular ROS, and in the regulation of several biological processes, including cell survival, proliferation and inflammation [14–16]. ATO enhances HMOX1 production in many cell systems, mostly via ROS-stimulated signaling pathways [14, 16–18]. Many evidences in the literature support an anti-apoptotic and protective role for HMOX1 in a variety of injury models [19–21]. However, it has also been described that these protective properties are restricted to a rather narrow threshold of overexpression [22], and that HMOX1 is neither exclusively cytoprotective nor exclusively cytotoxic [15].

CLL cells display intrinsic high levels of ROS/oxidative stress and upregulated HMOX1 expression, compared to normal B cells and these characteristics could constitute therapeutic targets [23, 24]. To help develop these strategies, a better knowledge of the role of HMOX1 in CLL as well as of the molecular pathways elicited by clinically relevant concentrations of ATO is needed. To address these issues, in the present report we have performed gene expression and functional analyses on CLL cells treated with ATO. We show that, by modulating the p38 MAPK signaling pathway, HMOX1 downregulates MMP-9 expression and contributes to ATO-induced cytotoxicity.

## RESULTS

### Gene expression profile triggered by ATO in CLL cells

**Figure 1:**
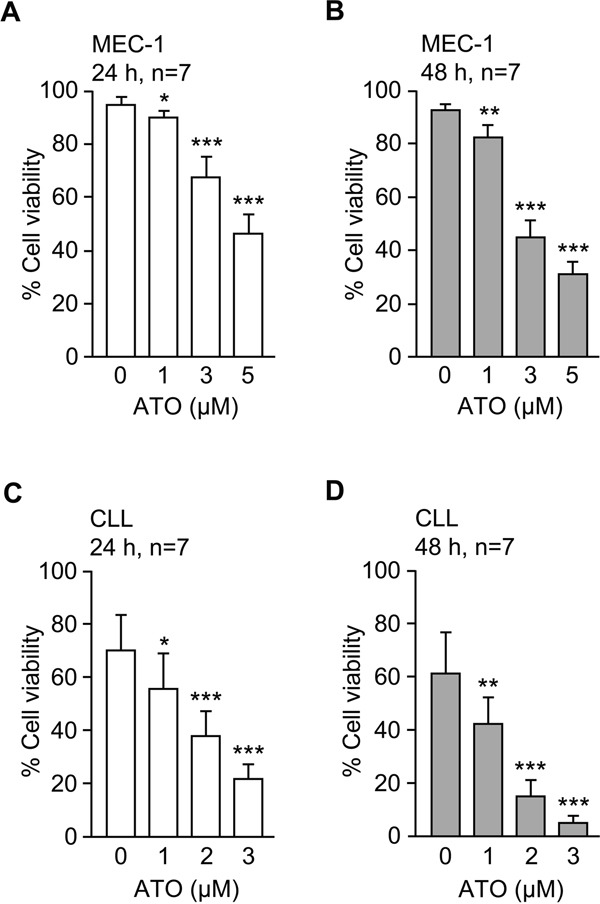
ATO efficiently induces apoptosis of MEC-1 and primary CLL cells 1.5 x 10^5^ MEC-1 cells in IMDM/0.1%FBS **A, B.** or 2 x 10^5^ CLL cells in RPMI/0.1%FBS **C, D.** were cultured with or without the indicated concentrations of ATO for 24 h (A, C) or 48 h (B, D). Cell viability was determined by flow cytometry, using FITC-Annexin V and PI. *P ≤ 0.05; **P ≤ 0.01; ***P ≤ 0.001.

To gain further insight into the mechanisms underlying the induction of CLL cell apoptosis by ATO we analyzed the gene expression profile upon treatment with this agent. The MEC-1 cell line (CLL-derived) was used for this purpose. To first determine the optimal conditions for the gene expression study, MEC-1 cells were incubated with several concentrations of ATO and their viability measured by flow cytometry after 24 and 48 h. As shown in Figure 1 and in agreement with our previous results [11], ATO decreased the viability of MEC-1 cells in a dose-dependent manner, after 24 (Figure 1A) or 48 h (Figure 1B). After 24 h of treatment with 5 μM ATO, approximately 50% of MEC-1 cells remained viable and these conditions were chosen for subsequent gene expression analyses. A similar dose-response effect was also observed for primary CLL cells, albeit ATO was more effective in this case and was used up to 3 μM to avoid excessive cell death (Figure 1C, 1D).

**Figure 2:**
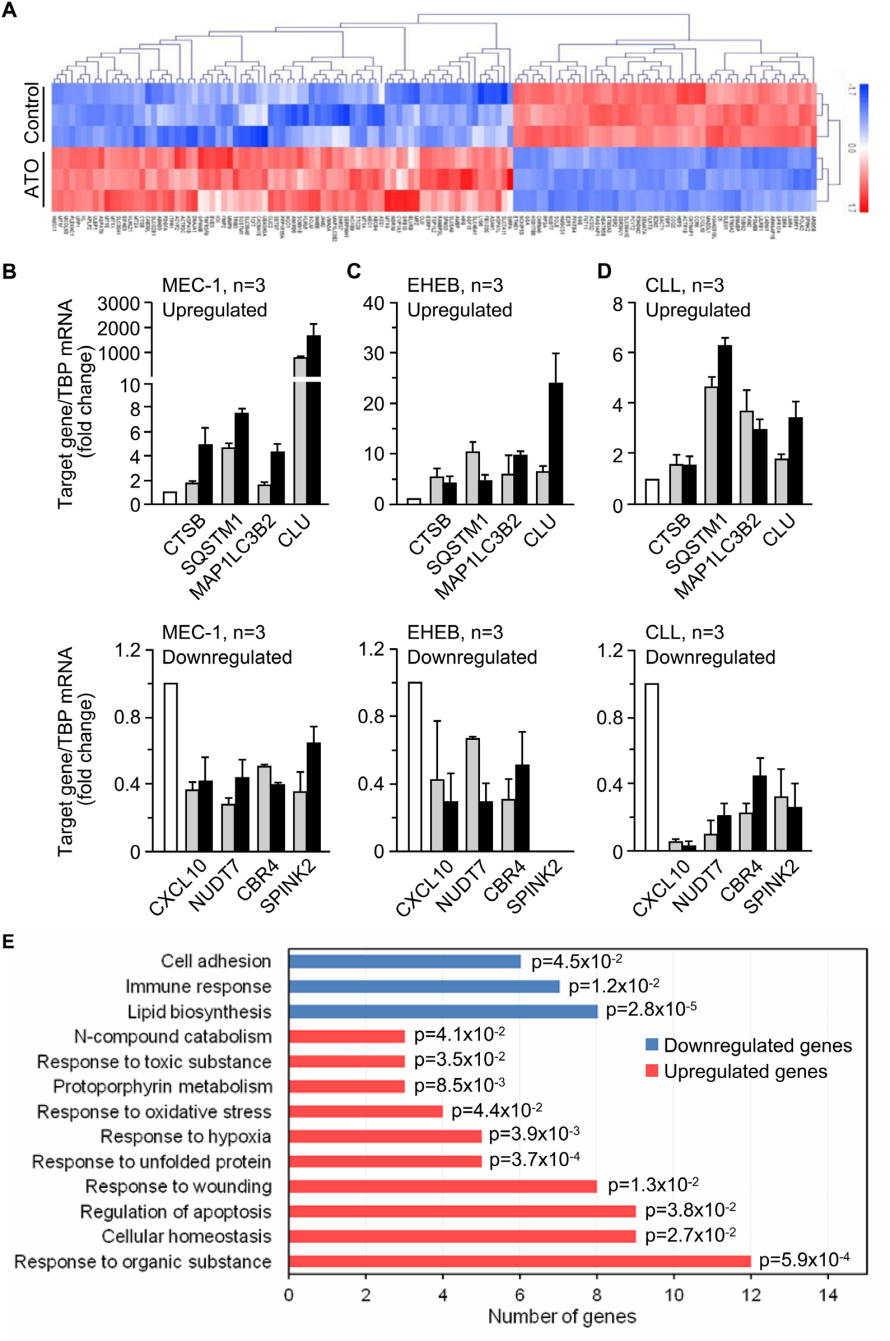
Gene expression profile triggered by ATO in CLL cells **A.** Heat map representing color-coded expression levels of 131 differently expressed genes (up- or down-regulated ≥ 2-fold and encoding characterized proteins) in ATO-treated vs untreated cells. Non-supervised hierarchical clustering of genes and samples is shown. **B-D.** qPCR validation of selected genes in MEC-1 (B), EHEB (C) and primary CLL (D) cells treated or not with ATO for 24 h. TBP expression was used as an internal control and normalized average values are shown. White columns, control; grey columns, 3 μM (MEC-1, EHEB) or 2 μM (CLL) ATO; black columns, 5 μM (MEC-1, EHEB) or 3 μM (CLL) ATO. All data shown are statistically significant (p-value ≤ 0.05). **E.** Functional annotation of the 131 genes shown in (A) using the Biological Process (BP)_FAT category of Gene Ontology and the DAVID database. p-values associated to each biological process are shown.

Initial data from gene microarray analyses of MEC-1 cells rendered a total of 738 differentially expressed genes (384 downregulated, 354 upregulated) between control and ATO-treated cells (GEO ID: GSE78207; website: http://www.ncbi.nlm.nih.gov/geo/). From these, we selected genes whose expression change was ≥ 2-fold, resulting in 148 genes (62 downregulated and 86 upregulated). Upon a second filtering process (elimination of genes encoding uncharacterized proteins or transcripts with little or no evidence at the protein level, pseudogenes and genes encoding Y RNA), a total of 131 differentially expressed genes (52 downregulated, 79 upregulated) were obtained (Figure 2A and Supplementary Table S1).

To validate the microarray results we selected several upregulated or downregulated genes and analyzed them by qPCR in MEC-1 cells. The four selected upregulated genes *(CTSB, SQSTM1, MAP1LC3B2, CLU)* were related to processes involving cell death (Supplementay Tables S1 and S2). For downregulated genes we randomly selected four genes among the most downregulated by ATO (*CXCL10, NUDT7, CBR4, SPINK2*) (Supplementary Tables S1 and S2). qPCR analyses confirmed the up- or downregulation, respectively, of the selected genes, in response to the two concentrations of ATO used, 3 and 5 μM (Figure 2B). Additionally, and to confirm that the results obtained in the microarray analysis reflected a general response of CLL cells to ATO, we validated the same selected genes on the EHEB cell line, also of CLL origin [25]. In initial analyses, ATO decreased the viability of EHEB cells in a dose-dependent manner, yielding 51.8% and 27.6% viable cells after 24 h treatment with 3 and 5 μM ATO, respectively (Supplementary Figure S1). Analyses by qPCR confirmed the upregulation of *CTSB, SQSTM1, MAP1LC3B2, and CLU,* and the down-regulation of *CXCL10, NUDT7,* and *CBR4 genes* on EHEB cells treated with ATO (Figure 2C), confirming the results obtained for MEC-1 cells. The *SPINK2* gene was not detected on EHEB cells.

To further validate the results with the cell lines we performed similar qPCR analyses using primary CLL cells from patients, treated or not with 2 or 3 μM ATO for 24 h. Figure 2D shows that the selected genes were also differentially regulated in primary CLL cells in response to ATO with respect to control cells. Altogether, and in spite of the not surprising fold-change differences in gene regulation among different cell types, the qPCR results confirmed the data of the microarray analyses and established that the observed gene expression profile was a general response of CLL cells.

### Functional classification of the differentially regulated genes by ATO

Having validated the microarray data we carried out functional analyses of the 131 genes displayed in Figure 2A and Supplementary Table S1, using the DAVID database and the biological process (BP_FAT) category of Gene Ontology. Upon discarding non-significantly enriched processes, these analyses revealed that ATO downregulated genes mainly involved in lipid metabolism, immune response and cell adhesion (Figure 2E). The significantly upregulated genes had roles in the response to oxidative stress, unfolded proteins, hypoxia, organic and toxic substances, and regulation of apoptosis, among others (Figure 2E). The specific genes included in these biological processes and their respective expression values (fold-change) are listed in Supplementary Table S2.

**Figure 3:**
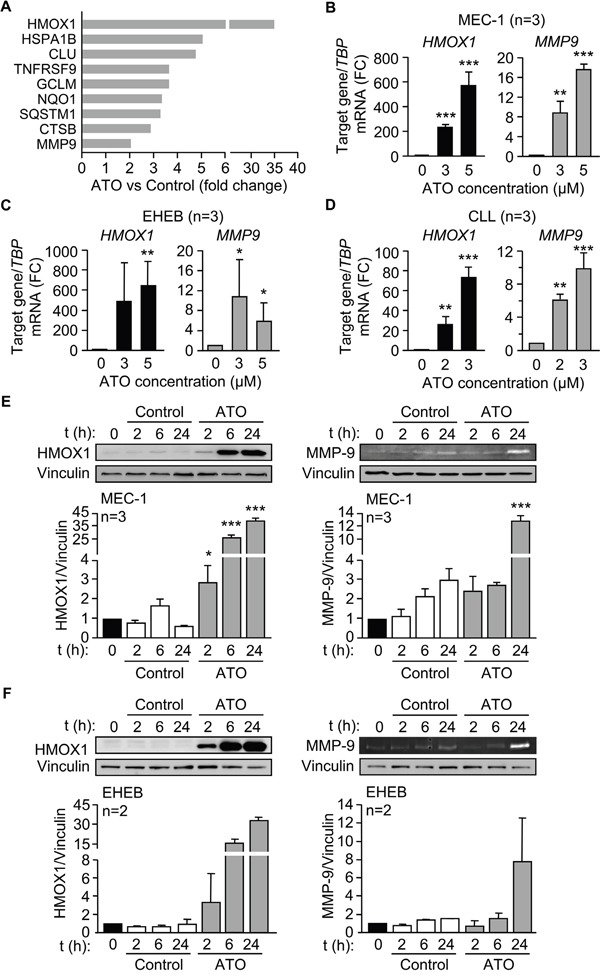
ATO induces HMOX1 and MMP-9 expression in CLL cells **A.** Bar graph representing the expression levels of the 9 differentially expressed genes involved in the regulation of apoptosis. **B-D.** 3-5 x 10^6^ MEC-1 cells in IMDM/0.1% FBS (B), 3-5 x 10^6^ EHEB cells in RPMI/0.1%FBS (C), or 10-15 x 10^6^ primary CLL cells in RPMI/0.1%FBS (D) were treated with the indicated concentrations of ATO for 24 h, and *MMP9* and *HMOX1* mRNA expression was analyzed by qPCR using TBP as internal control. Normalized average values are shown. **E-F.** 3-5 x 10^6^ MEC-1 (E) or EHEB (F) cells were treated or not with 5 μM ATO for the indicated times and analyzed by Western blotting (cell lysates) and gelatin zymography (concentrated conditioned media). FC, fold change; *P ≤ 0.05; **P ≤ 0.01; ***P ≤ 0.001, compared to their corresponding controls at each time point.

Because the main effect of ATO on CLL cells is the induction of apoptosis (Figure 1A, 1B and refs [8–10]) we focused on the 9 differentially upregulated genes involved in the regulation of this process (Supplementary Table S2). The expression levels of these genes are graphically represented in Figure 3A. The most upregulated gene by ATO was *HMOX1* (35-fold change), in agreement with the strong induction of ROS and oxidative stress caused by ATO in CLL and other cell types [10, 13, 26]. Another gene upregulated in this analysis was *MMP9*, confirming our previous report and the role of MMP-9 in the apoptotic response of CLL cells to ATO [11]. Therefore, we focused subsequent studies on HMOX1 (the most upregulated gene) and on MMP-9 (with known functions in CLL).

We first validated the above results at the gene and protein level. qPCR analyses clearly demonstrated that *HMOX1 and MMP9* expression was significantly increased by treatment of MEC-1 cells with either 3 or 5 μM ATO (Figure 3B). Moreover, *HMOX1 and MMP9* were also significantly upregulated upon incubation of EHEB cells with 3 or 5 μM ATO (Figure 3C) and of primary CLL cells with 2 or 3 μM ATO (Figure 3D), thus confirming the results obtained on MEC-1 cells.

To determine whether ATO also regulated HMOX1 and MMP-9 proteins, MEC-1 cells were treated with 5 μM ATO for various times and cell lysates analyzed by Western blotting. Figure 3E shows that the levels of HMOX1 were very low in control cells, in agreement with its inducible character, but significantly increased after 2 h of exposure to ATO, being even higher after 24 h of treatment. Gelatin zymography analysis of the concentrated conditioned medium of the same cells indicated that MMP-9 was also significantly induced after 24 h of ATO treatment (Figure 3E). Likewise, treatment of EHEB cells with ATO clearly increased HMOX1 after 2, 6, and 24 h (Figure 3F). The amount of MMP-9 secreted into the medium, measured by gelatin zymography, was also increased after 24 h of cell exposure to ATO (Figure 3F), confirming that EHEB and MEC-1 cells behaved similarly.

### ATO regulates MMP-9 expression in CLL cells via the p38 MAPK/c-jun signaling pathway

**Figure 4:**
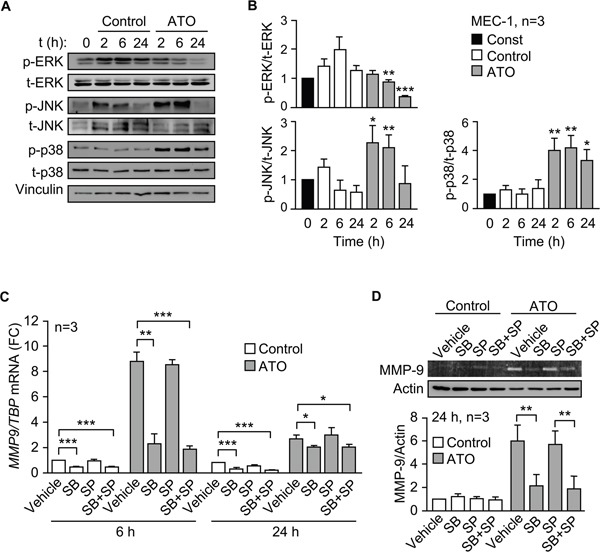
ATO upregulates MMP-9 in CLL cells via p38 MAPK activation **A, B.** 3-5 x 10^6^ MEC-1 cells in IMDM/0.1% FBS were incubated with or without 5 μM ATO for the indicated times and cell lysates analyzed by Western blotting. A representative experiment (A) and the quantitation of the three experiments performed (B), are shown. **C.** 3-5 x 10^6^ MEC-1 cells in IMDM/0.1% FBS were incubated with or without SB203580 (SB, 10 μM), SP600125 (SP, 5 μM) or both. After 1 h at 37°C, 5 μM ATO was added or not and cells further incubated for 6 and 24 h. Total RNA was extracted and *MMP9* mRNA levels analyzed by qPCR using TBP as internal control. Normalized average values are shown. FC, fold change. **D.** The conditioned medium of the same cells used in (C) was collected after 24 h and analyzed by gelatin zymography. The results from one representative experiment and the quantitation of the three experiments performed, after normalizing control values to 1, are shown. *P ≤ 0.05; **P ≤ 0.01; ***P ≤ 0.001, compared to their corresponding controls at each time point.

To study the mechanism involved in the regulation of MMP-9 by ATO we first analyzed the possible activation of relevant kinases. Because we have shown that ATO inhibits Akt phosphorylation and activates JNK [10], we focused the analysis on members of the MAPK family. ATO significantly decreased ERK1/2 phosphorylation after 6 h of treatment compared to control values. This was not due to apoptosis, since at this time apoptosis was <1%, determined by FITC-Annexin V/PI and flow cytometry (not shown). Phospho-ERK1/2 remained low after 24 h of exposure to ATO (Figure 4A, 4B). Because of these results, ERK1/2 was disregarded as a possible candidate and was not further studied. As previously observed in primary CLL cells [10], ATO increased JNK phosphorylation in MEC-1 cells after 2 and 6 h of treatment, then declining (Figure 4A, 4B). p38 MAPK was also clearly activated by ATO after 2 h and remained significantly phosphorylated after 24 h of treatment (Figure 4A, 4B).

To then establish whether JNK and/or p38 MAPK regulated MMP-9 expression, we inhibited these kinases and measured *MMP9* mRNA by qPCR. Figure 4C shows that, in the presence of ATO, the p38 MAPK inhibitor SB203580 significantly reduced (3.8-fold) *MMP9* mRNA levels after 6 h, while the JNK inhibitor SP600125 did not. The combination of both inhibitors did not significantly increase the effect of SB203580, which was still significant after 24 h of exposure to ATO (Figure 4C). The basal expression of *MMP9* mRNA in the absence of ATO, albeit low, was also significantly reduced by the p38 MAPK inhibitor, both after 6 and 24 h (Figure 4C). Additionally, gelatin zymography analyses demonstrated that SB203580, but not SP600125, also significantly diminished the levels of MMP-9 secreted into the conditioned medium after 24 h of cell treatment with ATO (Figure 4D). In the absence of this agent, the expression of MMP-9 was very low and differences could not be observed (Figure 4D).

**Figure 5:**
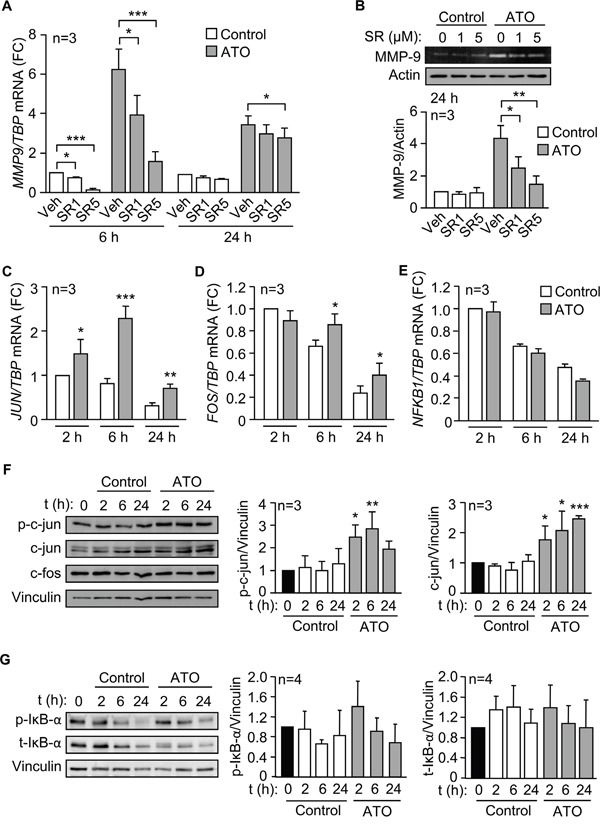
The AP-1 transcription factor is responsible for the upregulation of MMP-9 by ATO **A.** 3-5 x 10^6^ MEC-1 cells in IMDM/0.1% FBS were incubated with or without 1 or 5 μM SR11302 (SR). After 1 h at 37°C, 5 μM ATO was added or not and cells further incubated for 6 and 24 h. *MMP9* mRNA expression was analyzed by qPCR. **B.** The conditioned medium of MEC-1 cells, treated or not with the indicated inhibitors, was collected after 24 h, concentrated and analyzed by gelatin zymography. A representative experiment and the average quantitation of the three experiments performed are shown. **C-E.** MEC-1 cells were treated with ATO for the indicated times and *JUN* (C), *FOS* (D) and *NFKB1* expression (E) was analyzed by qPCR, using TBP as internal control. **F, G.** Western blotting analyses of the indicated proteins upon treatment of MEC-1 cells with ATO for the indicated times. A representative experiment and the average quantitation of the three (F) or four (G) experiments performed is shown. *P ≤ 0.05; **P ≤ 0.01; ***P ≤ 0.001, compared to their corresponding controls at each time point.

We next analyzed whether the transcription complex AP-1, a downstream effector of p38 MAPK and with several binding sites in the MMP-9 promoter [27, 28], was responsible for the upregulation of MMP-9 by ATO. To this end, we first studied the effect of the specific AP-1 inhibitor SR11302 on *MMP9* mRNA and protein expression. qPCR analyses showed that SR11302 significantly reduced *MMP9* expression in a dose-dependent manner, after 6 h of treatment with ATO, and the effect persisted after 24 h (Figure 5A). At the protein level, the AP-1 inhibitor also significantly inhibited secreted MMP-9 in a dose-dependent manner, measured by gelatin zymography after 24 h of ATO exposure (Figure 5B).

To confirm the major role of AP-1 in MMP-9 regulation we measured by qPCR whether the expression of c-jun and c-fos, the main components of the AP-1 complex, was modulated by ATO. *JUN* mRNA was significantly upregulated after 2 h of exposure to 5 μM ATO, with maximum levels after 6 h and still significantly higher than controls after 24 h (Figure 5C). ATO did not increase *FOS* mRNA after 2 h of treatment, but at longer times, *c-fos* values remained significantly higher in ATO-treated cells than in control cells (Figure 5D). In parallel analyses, we measured whether ATO increased the expression of NF-κB, another transcription factor activated by p38 MAPK and involved in the regulation of MMP-9 [27, 28]. qPCR analyses demonstrated that ATO did not upregulate *NFKB1* mRNA expression at any of the times studied (Figure 5E), in agreement with the reported inactivation of NF-κB upon ATO treatment of primary CLL cells [10].

We next studied whether ATO modulated c-jun and c-fos at the protein level. MEC-1 cells were treated or not with 5 μM ATO for the same times as above, lysed and analyzed by Western blotting. As shown in Figure 5F for a representative experiment and quantitated for the three experiments performed, c-jun phosphorylation significantly increased after 2 and 6 h of treatment with ATO (2.2 and 2.87 fold, respectively), compared to control cells. The expression of total c-jun was also significantly increased in response to ATO, remaining significantly elevated after 24 h of treatment (Figure 5F). These results indicated that the observed modulation of c-jun correlated well with the activation of p38 MAPK shown in Figure 4A, 4B. The expression of c-fos protein, however, was not modulated by ATO at the times studied (Figure 5F). To then determine whether the NF-κB protein was regulated by ATO we measured the phosphorylation and expression of the NF-κB-associated protein IκB, a reporter of NF-κB activation [29]. Figure 5G shows that ATO did not significantly modify either phosphorylated or total IκB, in agreement with the results shown in Figure 5E and suggesting a null or very low contribution of NF-κB to the regulation of MMP-9 by ATO.

**Figure 6:**
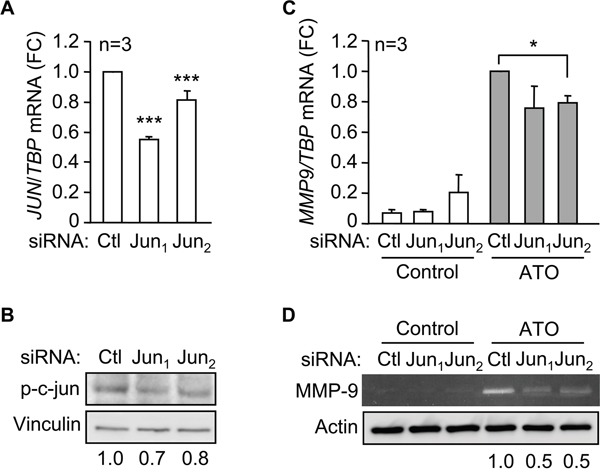
c-jun mediates the induction of MMP-9 by ATO 15 x 10^6^ MEC-1 cellswere nucleofected with two c-jun siRNAs (Jun_1_, Jun_2_) or a control siRNA (Ctl) for 16 h. *JUN* mRNA expression was analyzed by qPCR **A.** and phospho-c-jun expression by Western blot **B**. A representative analysis, as well as the quantitation of three experiments performed is shown. **C.** MEC-1 cells were nucleofected as in (A) and incubated with or without 5 μM ATO. *MMP9* mRNA expression was analyzed by qPCR after 6 h with or without ATO treatment. Normalized average values are shown. **D.** The conditioned media of siRNA transfected-MEC-1 cells, incubated with or without ATO for 48 h, was concentrated and analyzed by gelatin zymography, using actin as a loading control. A representative experiment is shown. Numbers indicate the normalized values of two different experiments. *P ≤ 0.05; ***^,^P ≤ 0.001.

To further establish the involvement of c-jun in MMP-9 upregulation in response to ATO, we transfected MEC-1 cells with two c-jun-specific siRNAs or with a control siRNA. This procedure reduced *JUN* mRNA expression 2- and 1.25-fold, respectively, for siJun_1_ and siJun_2_, determined by qPCR (Figure 6A). Western blotting analyses also confirmed the reduction of phospho-c-jun protein on cells transfected with siJun_1_ (1.4-fold) and siJun_2_ (1.25-fold) (Figure 6B). Subsequent qPCR analyses of c-jun silenced cells treated with ATO demonstrated a reduced expression of *MMP9* mRNA by 1.3-fold (siRNA_1_) and 1.25-fold (siRNA_2_), with respect to cells transfected with control siRNA (Figure 6C). Furthermore, the levels of secreted MMP-9 protein, measured by gelatin zymography after 48 h culture, were also reduced on c-jun siRNA transfected cells (Figure 6D). Altogether these results established that activation of p38 MAPK and c-jun were mainly responsible for the upregulation of MMP-9 in response to ATO.

### HMOX1 gene silencing upregulates p38 MAPK/c-jun activation and increases MMP-9 expression

**Figure 7:**
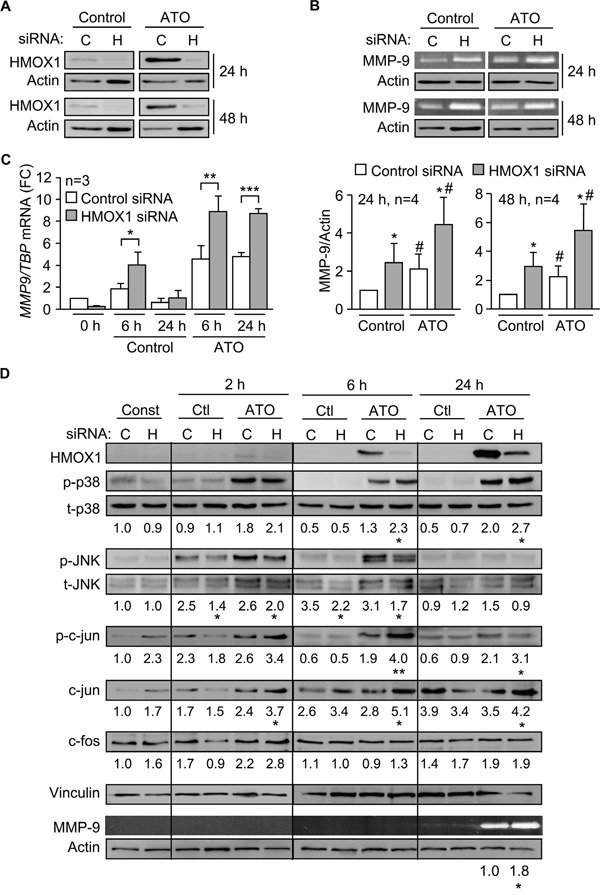
Gene silencing HMOX1 increases p38 MAPK/AP-1 activation and MMP-9 expression **A.** 15 x 10^6^ MEC-1 cells were nucleofected with HMOX1 (H) or control (C) siRNAs and incubated with or without 5 μM ATO for 24 h or 48 h. HMOX1 expression was analyzed by Western blotting. **B.** The conditioned media of the cells shown in (A) was concentrated and analyzed by gelatin zymography. A representative analysis, as well as the quantitation of the four experiments performed is shown. **C.** MEC-1 cells were nucleofected as in (A) and *MMP9* mRNA expression analyzed by qPCR after 6 and 24 h of ATO treatment. Normalized average values are shown. **D.** 15 x 10^6^ MEC-1 cells were nucleofected with HMOX1 (H) or control (C) siRNAs and treated with or without 5 μM ATO. At the indicated times, cells were lysed and analyzed by Western blotting. A representative experiment is shown. Numbers indicate the average values of three different experiments. Phosphorylated kinases were normalized with respect to their corresponding total protein; p-c-jun, c-jun, and c-fos were normalized with respect to vinculin. The conditioned media of these cells was analyzed by gelatin zymography, using actin as a loading control. *^, #^P ≤ 0.05; **^,^^##^P ≤ 0.01; ***^, ###^P ≤ 0.001. *, control siRNA vs HMOX1 siRNA; ^#^, control vs ATO.

Previous studies in other cell systems have demonstrated a relation between HMOX1 and certain MMPs [30–32]. We therefore studied whether HMOX1 regulated MMP-9 in CLL cells in response to ATO. To this end, we transfected MEC-1 cells with HMOX1 or control siRNAs and measured the expression of MMP-9. The efficiency of the transfection was confirmed by Western blotting, which showed that gene silencing HMOX1 reduced the already low levels of this protein in control cells (Figure 7A). In cells treated with ATO there was a 5.9-fold (average of 4 experiments) HMOX1 reduction after 24 h and a 5.2-fold reduction (average of 4 experiments) after 48 h, compared to their respective control siRNA (Figure 7A).

Gelatin zymography analyses indicated that gene silencing HMOX1 significantly upregulated secreted MMP-9, both in the absence or presence of ATO (Figure 7B), suggesting that HMOX1 negatively regulates MMP-9. To determine if this regulation was at the transcriptional level, we measured by qPCR the expression of *MMP9* mRNA on cells transfected with control- or HMOX1 siRNA. Figure 7C shows that *MMP9* mRNA was significantly higher in *HMOX1*-silenced cells than in control siRNA-transfected cells. For control cells (no ATO), upregulation of *MMP9* mRNA was clearly observed after 6 h. For cells treated with ATO, the maximal mRNA increase was also after 6 h, although significantly elevated *MMP9* mRNA levels persisted after 24 h of treatment (Figure 7C). Altogether these results indicated that HMOX1 transcriptionally regulates MMP-9 expression in CLL cells.

To next study whether HMOX1 regulated MMP-9 by influencing the p38 MAPK pathway we measured the activation of this kinase in MEC-1 cells transfected with control or *HMOX1* siRNAs. The phosphorylation of JNK was also measured in these experiments as a control for the specificity of the effect. HMOX1 was hardly detectable after 2 h of treatment with 5 μM ATO, but the HMOX1 siRNA effectively reduced ATO-induced HMOX1 expression after 6 and 24 h of treatment (shown in Figure 7D for a representative experiment out of the three performed). p38 MAPK phosphorylation was very low or undetectable in control cells, but in the presence of ATO gene silencing HMOX1 increased p38 MAPK activation 1.8- and 1.35-fold, respectively, after 6 and 24 h, compared to control siRNA (Figure 7D). In contrast, the phosphorylation of JNK was either unchanged or diminished by HMOX1 siRNA, after 2 or 6 h of ATO treatment, confirming the specificity towards p38 MAPK (Figure 7D).

Silencing HMOX1 also had a profound effect on the activation of c-jun, which increased 2.1-fold after 6 h of ATO treatment, and remained significantly higher than the corresponding control after 24 h (Figure 7D). The total expression of c-jun in ATO-treated cells was also significantly enhanced by gene silencing HMOX1 at all times studied (Figure 7D). c-fos expression also showed a moderate, but not statistically significant, increase after 2 h of ATO exposure in *HMOX1*-silenced cells, compared with control siRNA, with no further changes observed at longer times (Figure 7D). Parallel to the activation of p38 MAPK and c-jun, significant higher levels (1.8-fold increase) of secreted MMP-9 were also observed by gelatin zymography in *HMOX1*-silenced cells after 24 h of ATO treatment (Figure 7D).

### Overexpression of HMOX1 or modulation of its activity affects p38 MAPK/c-jun activation and MMP-9 expression

**Figure 8:**
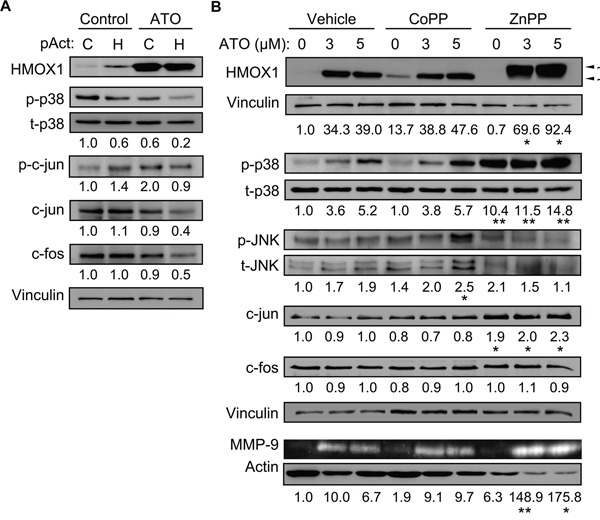
Overexpressing HMOX1 or modulating its activity affects p38 MAPK/c-jun activation and MMP-9 expression **A.** 10 x 10^6^ MEC-1 cells were nucleofected with CRISPR/dCas9 HMOX1 activation (pAct H) or control (pAct C) plasmids and incubated with or without 3 μM ATO. After 24 h, cell lysates were analyzed by Western blotting. Values represent the average of two different experiments. **B.** 3-5 x 10^6^ MEC-1 cells were incubated for 1 h with or without 3 μM CoPP or 2 μM ZnPP. 5 μM ATO was added or not and cells further incubated for 24 h. Cell lysates and conditioned medium were analyzed by Western blotting and gelatin zymography, respectively. The results of one representative experiment and the normalized average values of the three experiments performed are shown. *P ≤ 0.05; **P ≤ 0.01.

We next used the opposite strategy and overexpressed HMOX1 in MEC-1 cells using the CRISPR/dCas9 system. Western blotting analyses confirmed the expression of HMOX1 in cells transfected with the HMOX1 activation plasmid, even in the absence of ATO, confirming the functionality of the system (Figure 8A). Concomitant to HMOX1 overexpression, p38 MAPK phosphorylation decreased 1.7-fold in control cells and 3-fold in ATO-treated cells (Figure 8A). The phosphorylation and the total levels of c-jun also decreased (2.2-fold each) in HMOX1-transfected cells treated with ATO (Figure 8A). Downregulation of c-fos was also observed under these conditions (Figure 8A).

To further confirm the above results, we studied the effect of the HMOX1 inductor/activator CoPP and the HMOX1 inhibitor ZnPP on the activation of p38 MAPK and c-jun. After preliminary experiments, 3 μM CoPP and 2 μM ZnPP were chosen for the study. The activity of CoPP and ZnPP on HMOX1 was first confirmed by Western blotting analyses. As shown in Figure 8B for a representative experiment out of three performed, HMOX1 was undetectable in basal conditions but readily induced by both concentrations of ATO in control cells. In the absence of ATO, CoPP induced HMOX1 expression, confirming the activity of this compound. The combination of ATO and CoPP did not significantly increased HMOX1 expression, likely reflecting that maximal induction had been already achieved (Figure 8B). ZnPP by itself did not induce HMOX1 expression (Figure 8B). However, when combined with ATO, ZnPP had a dual effect: 1) it potentiated the increase on HMOX1 expression induced by ATO, in agreement with the reported role of ZnPP as an inducer of HMOX1 and an inhibitor of its activity [33, 34]; 2) it affected HMOX1 mobility in gels, suggesting that besides inhibiting HMOX1 activity, ZnPP binding induced a conformational change in HMOX1, further confirming the activity of this compound (Figure 8B).

CoPP did not significantly affect the induction of p38 MAPK phosphorylation by ATO, likely for the reasons mentioned above (Figure 8B). However, ZnPP significantly increased p38 MAPK phosphorylation by itself and both concentrations of ATO further enhanced this effect (Figure 8B), confirming the results obtained with the *HMOX1* siRNA. In contrast, JNK phosphorylation decreased in cells treated with ZnPP in the presence of ATO, with respect to the corresponding controls (Figure 8B). CoPP had no significant effect on c-jun expression, but ZnPP significantly increased (nearly 2-fold) c-jun, to similar levels with or without ATO (Figure 8B). These compounds did not change c-fos expression (Figure 8B). Parallel analyses by gelatin zymography indicated that CoPP was not able to significantly increase the amount of secreted MMP-9 and did not modify the levels induced by ATO (Figure 8B). However, ZnPP significantly increased the effect of ATO on MMP-9 production (Figure 8B). Altogether these results established that HMOX1 downregulates MMP-9 expression by interfering with activation of the p38 MAPK/c-jun signaling pathway.

### HMOX1 enhances the apoptotic effect of ATO in CLL cells

**Figure 9:**
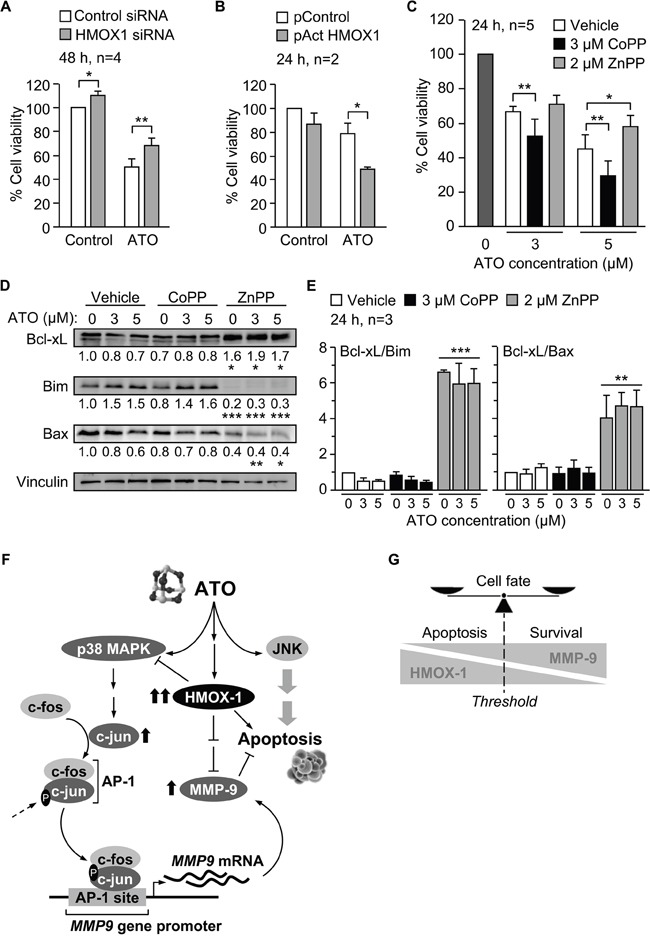
Pro-apoptotic role of HMOX1 in CLL cells in response to ATO **A.** 10-15 x 10^6^ MEC-1 cells were nucleofected with HMOX1 or control siRNAs and treated with or without 5 μM ATO for 48 h. Cell viability was analyzed by flow cytometry. **B.** 10 x 10^6^ MEC-1 cells were nucleofected with CRISPR/dCas9 HMOX1 or control plasmid and treated with or without 3 μM ATO for 24 h. Cell viability was determined as in (A). **C.** 3-5 x 10^6^ MEC-1 cells were incubated with vehicle or the indicated compounds and treated with the indicated concentrations of ATO. After 24 h, cell viability was determined as explained in (A). **D.** MEC-1 cells, treated or not with CoPP or ZnPP, were incubated with the indicated concentrations of ATO for 24 h and analyzed by Western blotting. Numbers indicate the normalized average values (protein/vinculin) from the three cases analyzed. **E.** Average quantitation of the Bcl-xL/Bim and Bcl-xL/Bax ratios. *P ≤ 0.05; **P ≤ 0.01; ***P ≤ 0.01. **F.** Schematic representation of the identified mechanisms of MMP-9 modulation by HMOX1 in CLL cells. ATO induces MMP-9 thought the activation of p38 MAPK/AP-1 pathway. ATO also induces the expression of HMOX1, which in turn transcriptionally downregulates MMP-9 by inhibiting p38 MAPK. **G.** Schematic drawing depicting how the levels of HMOX1 and MMP-9 may determine the cell fate. Within a particular threshold, high levels of HMOX1 and low levels MMP-9 would favor apoptosis, while high levels of MMP-9 and lower levels of HMOX1 would support cell survival.

Previous studies in several cell types have described an anti-apoptotic role for HMOX1 in response to ATO [35–37]. To determine whether HMOX1 influenced the apoptotic action of ATO in CLL cells, we first measured the viability of cells transfected with control or HMOX1 siRNAs. No significant differences were observed between control and HMOX-1-silenced cells after 24 h (not shown). However, after 48 h of culture, the viability of cells transfected with HMOX1 siRNA was significantly higher than when transfected with control siRNA (values normalized to 100), both in the absence or presence of ATO (1.1- and 1.4-fold increase, respectively) (Figure 9A). In agreement with these results, the viability of cells transfected with the HMOX1 activation plasmid was lower than in cells transfected with control plasmid, both in the absence, but particularly, in the presence of ATO (1.2- and 1.6-fold decrease, respectively) (Figure 9B).

We also studied the effect of modulating HMOX1 activity with CoPP or ZnPP, alone or combined with various concentrations of ATO, on cell viability. The viability of cells incubated in the absence of ATO was normalized to 100 in all cases. 3 μM CoPP alone did not decrease cell viability (98%, not shown), but when combined with ATO enhanced the apoptotic effect of this agent at all concentrations tested (Supplementary Figure S2A). Indeed, combination index analyses using the CompuSyn software indicated that the interaction of CoPP and ATO was synergistic for all ATO concentrations, except for 8 μM, which was additive (Supplementary Figure S2A).

Similar analyses were carried out with the HMOX1 inhibitor ZnPP. In this case, 2 μM ZnPP alone induced 36% cell death (average of three experiments, not shown). When combined with ATO, the viability values obtained for 1 and 2 μM ATO were higher than 36%, indicating that ZnPP counteracted the apoptotic effect of ATO, as clearly observed at higher concentrations of ATO (Supplementary Figure S2B). Consistently, analyses of the combination index demonstrated an antagonistic interaction between ZnPP and ATO at all concentrations of ATO tested (Supplementary Figure S2B).

To confirm these results, we measured the viability of cells cultured in the absence or presence of 3 μM CoPP or 2 μM ZnPP and with or without 3 or 5 μM ATO, the concentrations used throughout our study. After 24 h culture in the absence of ATO the average (n=5) viability values were 75.3% (vehicle), 77% (+3 μM CoPP), and 64% (+2 μM ZnPP), and were normalized to 100. The combination of CoPP with either 3 or 5 μM ATO significantly reduced cell viability, compared to control cells (Figure 9C). ZnPP had the opposite effect, inducing a moderate increase in cell viability when combined with 3 μM ATO but a significant increase upon combination with 5 μM ATO (Figure 9C).

To further establish the role of HMOX1 in the apoptosis of CLL cells induced by ATO we analyzed whether modulation of HMOX1 activity by CoPP and ZnPP affected the expression of Bcl-2 family proteins. As observed above for the regulation of p38 MAPK and c-jun (Figure 8B), CoPP did not significantly modify the levels of the anti-apoptotic protein Bcl-xL or the proapoptotic proteins Bim and Bax, with respect to controls (Figure 9D). However, inhibition of HMOX1 function by ZnPP significantly increased Bcl-xL expression and decreased the levels of Bim and Bax (Figure 9D). Other proteins of this family (Bcl-2, Mcl-1 and Noxa) were not significantly modulated by ZnPP (not shown). Moreover, and because regulation of survival/apoptosis depends on the balance of anti-apoptotic versus pro-apoptotic Bcl-2 family proteins [38], we also measured the ratios Bcl-xL/Bim and Bcl-xL/Bax. Figure 9E shows that both ratios were significantly upregulated upon cell treatment with ZnPP, both in the absence or presence of ATO.

In summary, our results establish that p38 MAPK/c-jun signaling is responsible for the increase of MMP-9 by ATO, and suggest a central role for HMOX1 in the modulation of this pathway and in the apoptotic response of CLL cells to ATO. A schematic drawing depicting these HMOX1 functions is shown in Figure 9F. An additional representation of how the levels of HMOX1 and MMP-9 may determine cell fate (apoptosis vs survival) is shown in Figure 9G.

## DISCUSSION

To expand our knowledge on the apoptotic mechanism induced by ATO and its potential use in CLL therapy, we have studied the global gene expression profile elicited by this agent in CLL cells. Our major findings are: 1) Gene expression analysis reflects an antioxidant and defensive cellular response to ATO; 2) ATO strongly upregulates HMOX1 and, to a lesser extent, MMP-9; 3) The mechanism by which ATO upregulates MMP-9 is via activation of the p38 MAPK/AP-1 pathway; 4) HMOX1 interferes with p38 MAPK/AP-1 activation and downregulates MMP-9; 5) HMOX1 exerts a pro-apoptotic role in CLL cells in response to ATO, synergistically enhancing the cytotoxicity of this agent.

Gene expression analyses by microarrays indicated that ATO regulated many genes in CLL cells. Importantly, this gene modulation was observed at clinically relevant concentrations of ATO [39, 40]. Most of the upregulated genes were allocated to the response to oxidative stress, endoplasmic reticulum stress (unfolded protein response), and organic/toxic substances, consistent with the induction of an antioxidant and defensive cell response. Accordingly, the most upregulated genes were *HMOX1* and genes encoding several members of the metallothionein family (*MT1F, MT1E, MT1G, and MT1X*), with well-established antioxidant and detoxification functions, respectively [41, 42]. Induction of *HMOX1* in response to oxidative stress, elicited for example by arsenical-based compounds, has been reported in several cell systems [14, 17, 43, 44]. The role of HMOX1 in CLL is not well known and our present results are the first to show that ATO strongly upregulates HMOX1 (gene and protein) in CLL cells. These results are particularly relevant in view of recent studies demonstrating that, besides its antioxidant function, HMOX1 is involved in mitochondrial biogenesis in CLL and could represent a potential therapeutic target [23].

Another important molecule in CLL is MMP-9 [11, 45–47] and our current analyses showed that MMP-9 expression was also upregulated by ATO, both at the gene and protein level. These findings confirm our previous report showing that ATO increases membrane-associated MMP-9 in CLL cells [11]. The molecular mechanism underlying this effect was not previously studied, and we have addressed this in the present report. Our results clearly show that ATO upregulates MMP-9 via activation of the p38 MAPK. Although ATO also activated JNK in CLL cells, inhibition of this kinase did not decrease MMP-9 induction, ruling out its role in the upregulation of MMP-9 in these conditions. In other cell systems, p38 MAPK mediates MMP-9 upregulation in response to several stimuli, including TNF-α and SDF-1/CXCR7 in bladder and ovarian, respectively, carcinoma cells [48, 49], and clusterin/thrombospondin-1 in platelet-stimulated colon cancer [50]. In CLL cells, constitutive activation of p38 MAPK was required for MMP-9 production in CLL-stromal cell co-cultures, as well as for the survival of CLL cells in this system [51]. Other authors have shown that p38 MAPK activity is associated to the induction of apoptosis in CLL cells [52, 53], indicating the dual role of this kinase in the survival of CLL cells.

Further analyses on the p38 MAPK signaling pathway demonstrated that AP-1 is the main transcription factor responsible for the induction of MMP-9 in response to ATO. This is consistent with the reported activation of AP-1 by p38 and other MAPK [54] and with the presence of several binding sites for AP-1 in the MMP-9 promoter [27, 28]. Indeed, the distal and proximal AP-1 binding sites have been shown to contribute to the transcriptional induction of MMP-9 in HT1080 cells, in response to various stimuli [55]. Although NF-κB is also known to induce MMP-9 transcription [27, 28], our results indicate that its contribution to the upregulation of MMP-9 by ATO, if any, is minimal, confirming our previous report showing that ATO inactivates NF-κB in primary CLL cells [10]. We have therefore identified the molecular mechanism by which ATO induces MMP-9 expression in CLL cells.

Previous reports in other cell systems have shown a connection between HMOX1 and the expression of several MMPs. For example, HMOX1 downregulated the expression and secretion of MMP-1 in chondrocytes [30] or plasma of patients with pulmonary tuberculosis [31]. Reduction of MMP-9 expression by HMOX1 was also observed in breast cancer cells [32]. We now show that HMOX1 transcriptionally downregulates MMP-9 production in CLL cells by interfering with p38 MAPK/AP-1 activation. The following evidences strongly support this conclusion: 1) gene silencing HMOX1 or inhibiting its activity with ZnPP resulted in increased p38 MAPK/AP-1 activation and MMP-9 production; 2) overexpression of HMOX1 or enhancement of its activity with CoPP decreased p38 MAPK activation. Additionally, these results are in agreement with a previous study showing that inhibition of HMOX1 in dendritic cells increases activation of the p38 MAPK signaling pathway [56]. The exact mechanism by which HMOX1 modulates p38 MAPK in CLL cells is not known, but probably involves some of the metabolites derived from HMOX1 function. In support of this, it has been demonstrated that carbon monoxide, a by-product of heme catabolism by HMOX1, mediates anti-inflamatory effects by regulating p38 and other MAPKs [57]. Therefore, by-products of HMOX1 can influence p38 MAPK activation and consequently, MMP-9 expression, as we observe. Based on our current results it is possible to speculate that the moderate increase in MMP-9 (2-fold), observed upon ATO treatment, could be due to the parallel repressive effect of HMOX1.

Besides regulating MMP-9 expression, our results show that HMOX1 enhances the cytotoxic effect of ATO in CLL cells in a synergistic manner, thus exerting a pro-apoptotic role in the response to this agent. Because we have previously shown that MMP-9 has an anti-apoptotic role in the presence of ATO (and other drugs) [11], downregulation of MMP-9 may be one of the mechanisms contributing to HMOX1 pro-apoptotic function. Our current findings differ from other studies showing a protective role for HMOX1 in other cellular systems [19–21, 58]. However, some evidences suggest that the protective properties of HMOX1 are restricted to a narrow threshold of expression and that HMOX1 may in fact have protective and cytotoxic functions, depending on the context [16, 22]. It has also been proposed that HMOX1 may display pro- and anti-oxidant properties [59], indirectly behaving as a pro- or anti-apoptotic protein. In this regard, and in agreement with our results, Hamamura et al. reported that induction of HMOX1 by cobalt protoporphyrin enhances the cytotoxic effect of bortezomib in T-cell leukemia cells [60]. Additionally, induction of HMOX1 via activation of the transcription factor Nrf2 by auranofin [24] or electrophilic compounds [61] increased the cytotoxic activity of these agents against CLL cells, confirming the apoptotic role of HMOX1.

In summary, our study is the first to report that HMOX1 negatively regulates MMP-9 expression in CLL cells in response to ATO, through modulation of the p38 MAPK/AP-1 signaling pathway. Additionally, modulation of HMOX1 expression or activity has a direct impact in the cytotoxic potential of ATO. The role of oxidative stress in CLL is beginning to draw attention as a possible target in this disease [23, 24]. Our current report reinforces these studies and indicates that the combination of ATO with HMOX1 modulators may represent an efficient alternative for the clinical treatment of this malignancy.

## MATERIALS AND METHODS

### Patients, cells and cell cultures

Approval was obtained from the CSIC Bioethics Review Board for these studies. Peripheral blood samples from the 8 CLL patients listed in Table 1 were obtained after informed consent, and B-lymphocytes were purified as described [11]. The MEC-1 and EHEB cell lines, established from CLL patients [62] were purchased from the German Collection of Microorganisms and Cell Cultures (DSMZ, Braunschweig, Germany) and cultured in IMDM/10% FBS (MEC-1) or RPMI/10% FBS (EHEB).

**Table 1:** Clinical characteristics of CLL patients

#Patient	Sex/Age^a^	Stage^b^	CD38/ZAP70^c^	IgH_v_ status^c^
1	F/72	C/IV	−/+	Mutated
2	M/70	B/II	+/+	Unmutated
3	M/65	A/I	−/−	Mutated
4	M/79	B/II	+/−	Unmutated
5	M/59	C/IV	+/+	Unmutated
6	F/73	A/II	−/−	Mutated
7	M/48	B/I	+/+	Unmutated
8	F/54	A/0	+/−	Unmutated

a M, male; F, female.

b Clinical stage according to references [1, 2].

c The coexpression of CD38 and ZAP-70, and IGHV gene mutational status have clinical prognostic value [1, 2].

### Antibodies and reagents

Rabbit polyclonal antibodies (RpAbs) to JNK (sc-571) and c-fos (sc-52), and mouse monoclonal Abs (mAbs) to c-jun (sc-166540), IκB-α (sc-1643) and HMOX1 (sc-136961) were from Santa Cruz Biotechnology (Santa Cruz, CA). mAbs to Vinculin (V9131) and β-actin (A5316) were from Sigma-Aldrich (St. Louis, MO). RpAbs to phospho-JNK (T183/y185), phospho-c-jun (S73), phospho-ERK1/2 (T202/Y204), phospho-p38 MAPK (T180/Y182) and p38 MAPK, and mAbs to ERK1/2 and phospho-IκB-α (S32/36) were from Cell Signaling Technology Inc. (Beverly, MA). Annexin V-FITC was from Immunostep (Salamanca, Spain). Propidium iodide (PI), MTT (3-(4,5-dimethylthiazolyl-2)-2,5-diphenyltetrazoliumbromide), arsenic trioxide (ATO), and the cobalt and zinc protoporphyrins [CoPP (HMOX1 activator) and ZnPP (HMOX1 inhibitor)] were from Sigma-Aldrich. SR11302 (AP-1 inhibitor) was from Santa Cruz Biotechnology. MAPK inhibitors SB203580 (p38) and SP600125 (JNK) were from Calbiochem (Darmstadt, Germany).

### Gene expression analysis by microarrays

5 x 10^6^ MEC-1 cells were cultured in IMDM/0.1% FBS with or without 5 μM ATO for 24 hours. Total RNA was extracted and purified using the RNeasy^®^ Mini kit (Qiagen, Limburg, Netherlands). Double-stranded cDNA and biotinylated cRNA were synthesized from 200 ng total RNA using the Ambion® WT Expression Kit (Thermo Fisher Scientific, MA, USA). Biotin-labeled cRNA was fragmented and hybridized to a GeneChip® Human Gene 1.0 ST Array (Affymetrix, Santa Clara, CA). Expression values were normalized and summarized using the Robust Multi-Array Average algorithm [63, 64]. Differential expression analyses in ATO-treated versus untreated samples were conducted by the Significance Analysis of Microarray method [65], setting the false discovery rate to 0.1 and considering a p value < 0.05 as statistically significant. Genes with significantly different expression (≥ 2-fold change) were manually filtered and the resulting group functionally annotated using the BP_FAT category of Gene Ontology (GO) and the Database for Annotation, Visualization and Integrated Discovery (DAVID, National Institute of Allergy and Infectious Diseases) v6.7. GO BP_FAT terms with an associated p value of ≤ 0.05 were considered significantly enriched. Clustering (non-supervised hierarchical) and heat maps were conducted with TIGR Multiexperiment Viewer v4.9, from the TM4 Software suite (Dana-Farber Cancer Institute, Boston, MA). The complete gene expression data sets have been deposited and are available online at the Gene Expression Omnibus repository (GEO ID: GSE78207).

### Quantitative PCR

Total RNA isolation and cDNA amplification were performed as described [11]. Quantitative PCR (qPCR) was performed using iQTM SYBR® Green Supermix (Bio-Rad Laboratories, Hercules, CA), and the primers listed in Supplementary Table S3. All assays were performed in triplicate and the results were normalized according to the expression levels of TBP and expressed using the ΔΔCT method for quantization.

### RNA interference experiments

The following siRNA sequences targeting human HMOX1 or c-jun were used: HMOX1: sense 5′-CUGUGUCCCUCUCUCUGGA[dT][dT]-3′; siJun_1_: 5′-GAUGGAAACGACCUUCUAU[dT][dT]-3′; siJun_2_: 5′-CCUUCUAUGACGAUGCCCU[dT][dT]-3′. The siRNA sequence for negative control was: sense 5′-AUUGUA UGCGAUCGCAGACdTdT-3′. siRNA duplexes were verified to be specific for their targets by Blast search against the human genome and were custom-made by Sigma-Aldrich. 15 x 10^6^ MEC-1 cells were nucleofected with 4 μM siRNAs using solution V and program T-02 (Amaxa, Cologne, Germany), and assayed 16 h after transfection. Efficiency of the transfection was confirmed by qPCR and Western blotting.

### Overexpression and regulation of HMOX1 activity

The CRISPR/dCas9 system for HMOX1 expression (HMOX1 CRISPR Activation Plasmid) and the corresponding control were purchased from Santa Cruz Biotechnology. 10 x 10^6^ MEC-1 cells were nucleofected with 5 μg plasmid system (Control or HMOX-1) as above, and assayed 16 h after transfection. Efficiency of the transfection was confirmed by Western blotting.

For regulation of HMOX1 activity, 1 x 10^6^ MEC-1 cells/ml in IMDM/0.1% FBS were incubated with 2 μM ZnPP, 3 μM CoPP or vehicle for 1 h at 37°C. ATO was added and cells further incubated for 24 h. The modulation of HMOX1 was confirmed by Western blotting.

### Analysis of cell viability

Primary CLL cells in RPMI/0.1% FBS, MEC-1 cells in IMDM/0.1% FBS, or EHEB cells in RPMI/0.1% FBS were incubated with ATO for 24-48 h and cell viability/apoptosis was determined on a Coulter Epics XL flow cytometer (Beckman Coulter, Fullerton, CA), using FITC-Annexin V and propidium iodide (PI). For MTT assays, 0.75 x 10^5^ MEC-1 cells in IMDM/0.1% FBS were incubated with 50 μg MTT for 4 h in the dark. The blue MTT formazan precipitate was dissolved in isopropanol-HCl (24:1) and the absorbance at 540 nm was determined on a Multiskan Bichromatic microplate reader (Labsystems, Helsinki, Finland). Drug interaction analyses were performed using the CompuSyn software (BioSoft, Cambridge, UK), which allows the calculation of the combination index (CI) values based on the algorithm of Chou and Talalay [66]. Combination index values < 1 indicate synergism, values < 1 (0.9-1.1) indicate additivity, and > 1, antagonism.

### Other methods

Western blotting, gelatin zymography and statistical analyses were performed exactly as described [11]. All values are expressed as means ± standard deviation, except for qPCR analyses where means ± standard error is shown.

## SUPPLEMENTARY FIGURES AND TABLES




