# Supplementary material for: Gene expression profile induced by arsenic trioxide in chronic lymphocytic leukemia cells reveals a central role for heme oxygenase-1 in apoptosis and regulation of matrix metalloproteinase-9
Source: Oncotarget. 2016 Nov 4;7(50):83359–77. doi: 10.18632/oncotarget.13091 (PMC5347775; doi:10.18632/oncotarget.13091)
Supplement: Supplementary file 1 [file oncotarget-07-83359-s001.pdf]

# Gene expression profile induced by arsenic trioxide in chronic lymphocytic leukemia cells reveals a central role for heme oxygenase-1 in apoptosis and regulation of matrix metalloproteinase-9

## SUPPLEMENTARY FIGURES AND TABLES

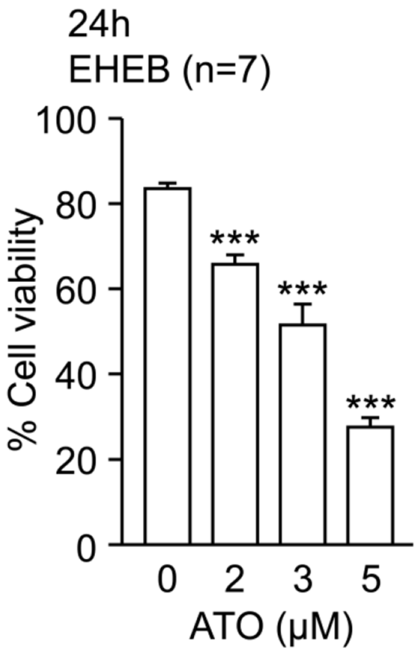


**Supplementary Figure S1: ATO induces apoptosis of EHEB cells.** 1.5 x $10^5$ EHEB cells in RPMI/0.1%FBS were cultured with or without the indicated concentrations of ATO. After 24 h, cell viability was determined by flow cytometry, using FITC-Annexin V (AnnV) and PI. ***$P \leq 0.001$.

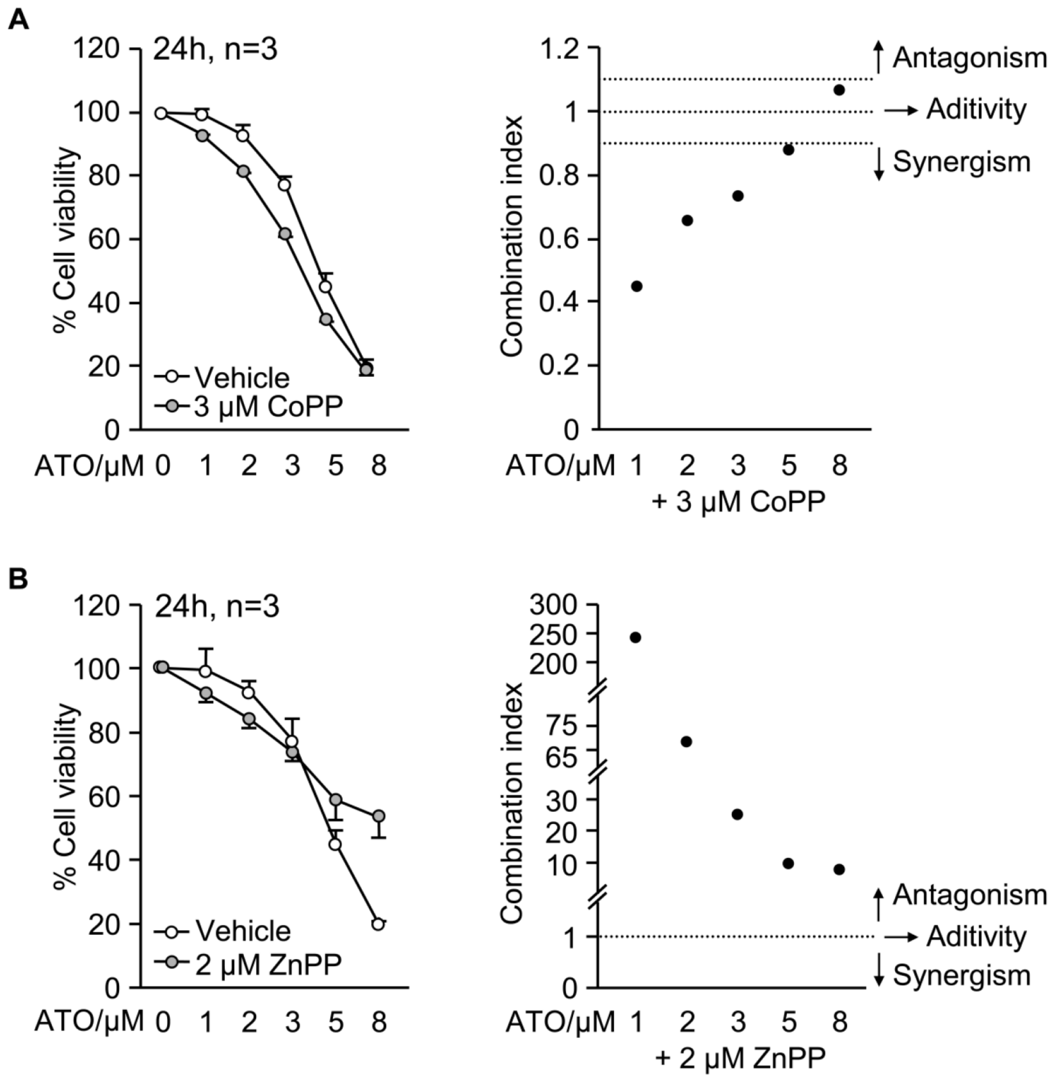


**Supplementary Figure S2: Effect of combining ATO with CoPP or ZnPP on MEC-1 cell viability.** 0.75 x $10^5$ MEC-1 cells were pre-incubated with or without 3 μM CoPP **A.** or 2 μM ZnPP **B.** for 1 h at 37°C. The indicated concentrations of ATO were added and cells further incubated for 24 h. Cell viability was determined by the MTT method. Each control (no ATO) was normalized to 100 and average values (n=3) are shown. Combination index (CI) values were calculated using the CompuSyn software (BioSoft, Cambridge, UK).

**Supplementary Table S1: Significantly modulated genes (52 downregulated, 79 upregulated) by ATO in MEC-1 cells corresponding to the heat map shown in Figure 2A**

See Supplementary File 1

**Supplementary Table S2: Biological process allocation of significantly regulated genes by ATO in MEC-1 cells, listed in alphabetical order within each category. See also Figure 2E**

See Supplementary File 1

**Supplementary Table S3: Oligonucleotide sequences used in the qPCR analyses**

| Gene name | | Oligonucleotide sequence |
|---|---|---|
| *CBR4* | *sense* | 5′- CATTGCCAGAAACCTGGAAG-3′ |
| | *antisense* | 5′-AGCTAAATGCCAAATGATCTCC-3′ |
| *CLU* | *sense* | 5′- GGGACCAGACGGTCTCAG-3′ |
| | *antisense* | 5′- CGTACTTACTTCCCTGATTGGAC-3′ |
| *CTSB* | *sense* | 5′- CAGCCACCCAGATGTAAGC-3′ |
| | *antisense* | 5′-GCCGGATCCTAGATCCAC-3′ |
| *FOS* | *sense* | 5′- ACTACCACTCACCCGCAGAC -3′ |
| | *antisense* | 5′- CCAGGTCCGTGCAGAAGT -3′ |
| *JUN* | *sense* | 5′-TTCTATGACGATGCCCTCAACGC -3′ |
| | *antisense* | 5′-GCTCTGTTTCAGGATCTTGGGGTTAC-3′ |
| *CXCL10* | *sense* | 5′- GAAAGCAGTTAGCAAGGAAAGGT-3′ |
| | *antisense* | 5′- GACATATACTCCATGTAGGGAAGTGA-3 |
| *HMOX1* | *sense* | 5′-GGGTGATAGAAGAGGCCAAGA-3′ |
| | *antisense* | 5′-AGCTCCTGCAACTCCTCAAA-3′ |
| *MAP1LC3B2* | *sense* | 5′- CGCACCTTCGAACAAAGAG-3′ |
| | *antisense* | 5′- CTCACCCTTGTATCGTTCTATTATCA-3′ |
| *MMP9* | *sense* | 5′-GAACCAATCTCACCGACAGG-3′ |
| | *antisense* | 5′-GCCACCCGAGTGTAACCATA-3′ |
| *NFKB1* | *sense* | 5′-CTGGCAGCTCTTCTCAAAGC -3′ |
| | *antisense* | 5′-TCCAGGTCATAGAGAGGCTCA -3′ |
| *NUDT7* | *sense* | 5′- CGTCCGGTCAGAGAAGCTA-3′ |
| | *antisense* | 5′- GGGTCACGCTTACCTCCAG-3′ |
| *SPINK2* | *sense* | 5′- GAGTGGCGCAGGTAACAGAC-3′ |
| | *antisense* | 5′- ACCAAATTGAGGGATCAGAGAG-3′ |
| *SQSTM1* | *sense* | 5′- AGCTGCCTTGTACCCACATC-3′ |
| | *antisense* | 5′- CAGAGAAGCCCATGGACAG-3′ |
| *TBP* | *sense* | 5′-CGGCTGTTTAACTTCGCTTC-3′ |
| | *antisense* | 5′-CACACGCCAAGAAACAGTGA-3′ |
