# Supplementary material for: Gene expression profile induced by arsenic trioxide in chronic lymphocytic leukemia cells reveals a central role for heme oxygenase-1 in apoptosis and regulation of matrix metalloproteinase-9
Source: Oncotarget. 2016 Nov 4;7(50):83359–77. doi: 10.18632/oncotarget.13091 (PMC5347775; doi:10.18632/oncotarget.13091)
Supplement: Supplementary file 2 [file oncotarget-07-83359-s002.docx]

Supplemental Table S1. Significantly modulated genes (52 downregulated, 79 upregulated) by ATO in MEC-1 cells corresponding to the heat map shown in Figure 2A.

| **Gene**  **name** | **Functional description** | **Fold**  **change** |
| --- | --- | --- |
| CXCL10 | chemokine (C-X-C motif) ligand 10 | -6.81 |
| CCR8 | chemokine (C-C motif) receptor 8 | -4.88 |
| DACT1 | dapper, antagonist of beta-catenin, homolog 1 (*Xenopus laevis*) | -4.26 |
| PFKFB4 | 6-phosphofructo-2-kinase/fructose-2,6-biphosphatase 4 | -4.22 |
| SLC39A10 | solute carrier family 39 (zinc transporter), member 10 | -3.76 |
| NUDT7 | nudix (nucleoside diphosphate linked moiety X)-type motif 7 | -3.05 |
| CBR4 | carbonyl reductase 4 | -3.04 |
| BTN3A2 | butyrophilin, subfamily 3, member A2 | -3.04 |
| HMGCS1 | 3-hydroxy-3-methylglutaryl-Coenzyme A synthase 1 (soluble) | -2.96 |
| PER2 | period homolog 2 (*Drosophila*) | -2.96 |
| SPINK2 | serine peptidase inhibitor, Kazal type 2 (acrosin-trypsin inhibitor) | -2.90 |
| CADM1 | cell adhesion molecule 1 | -2.88 |
| FUT11 | fucosyltransferase 11 (alpha (1,3) fucosyltransferase) | -2.88 |
| RPSA | 40S ribosomal protein SA | -2.83 |
| BZW2 | basic leucine zipper and W2 domains 2 | -2.73 |
| BTN3A3 | butyrophilin, subfamily 3, member A3 | -2.70 |
| FLRT3 | fibronectin leucine rich transmembrane protein 3 | -2.66 |
| C5 | complement component 5 | -2.64 |
| CHRNA6 | cholinergic receptor, nicotinic, alpha 6 | -2.61 |
| NCKIPSD | NCK interacting protein with SH3 domain | -2.55 |
| COQ2 | coenzyme Q2 homolog, prenyltransferase (yeast) | -2.51 |
| HEATR5B | HEAT repeat containing 5B | -2.49 |
| FDPS | farnesyl diphosphate synthase (farnesyl pyrophosphate synthetase, dimethylallyltranstransferase, geranyltranstransferase) | -2.46 |
| SEMA7A | semaphorin 7A, GPI membrane anchor | -2.41 |
| PSMB8 | proteasome (prosome, macropain) subunit, beta type, 8 (large multifunctional peptidase 7) | -2.39 |
| R3HDM2 | R3H domain containing 2 | -2.37 |
| GNE | glucosamine (UDP-N-acetyl)-2-epimerase/N-acetylmannosamine kinase | -2.32 |
| GPR174 | G protein-coupled receptor 174 | -2.30 |
| HSD17B8 | hydroxysteroid (17-beta) dehydrogenase 8 | -2.30 |
| E2F5 | E2F transcription factor 5, p130-binding | -2.30 |
| EDRF1 | erythroid differentiation-related factor 1 | -2.27 |
| DNMBP | dynamin binding protein | -2.26 |
| ARHGAP18 | Rho GTPase activating protein 18 | -2.26 |
| PCYT2 | phosphate cytidylyltransferase 2, ethanolamine | -2.19 |
| GDA | guanine deaminase | -2.18 |
| ACTR1B | ARP1 actin-related protein 1 homolog B, centractin beta (yeast) | -2.17 |
| ARID5B | AT rich interactive domain 5B (MRF1-like) | -2.16 |
| PAN2 | PAN2 poly(A) specific ribonuclease subunit homolog (*S. cerevisiae*) | -2.13 |
| RAG1AP1 | recombination activating gene 1 activating protein 1 | -2.09 |
| POLG2 | polymerase (DNA directed), gamma 2, accessory subunit | -2.07 |
| LILRB1 | leukocyte immunoglobulin-like receptor, subfamily B (with TM and ITIM domains), member 1 | -2.07 |
| H6PD | hexose-6-phosphate dehydrogenase (glucose 1-dehydrogenase) | -2.04 |
| SDR39U1 | short chain dehydrogenase/reductase family 39U, member 1 | -2.02 |
| DLEU1 | deleted in lymphocytic leukemia 1 (non-protein coding) | -2.01 |
| LGR4 | leucine-rich repeat-containing G protein-coupled receptor 4 | -2.01 |
| CNTNAP1 | contactin associated protein 1 | -2.01 |
| TUBG2 | tubulin, gamma 2 | -2.01 |
| MAD2L1 | MAD2 mitotic arrest deficient-like 1 (yeast) | -2.00 |
| KIAA0319L | KIAA0319-like | -1.99 |
| ACSS2 | acyl-CoA synthetase short-chain family member 2 | -1.99 |
| SQLE | squalene epoxidase | -1.98 |
| ETHE1 | ethylmalonic encephalopathy 1 | -1.98 |
| ANKRD6 | ankyrin repeat domain 6 | 2.02 |
| ASAH1 | N-acylsphingosine amidohydrolase (acid ceramidase) 1 | 2.03 |
| FBXO30 | F-box protein 30 | 2.04 |
| ACTR3C | ARP3 actin-related protein 3 homolog C (yeast) | 2.05 |
| MT1A | metallothionein 1A | 2.07 |
| MMP9 | matrix metallopeptidase 9 (gelatinase B, 92kDa gelatinase, 92kDa type IV collagenase) | 2.08 |
| CACNA1E | calcium channel, voltage-dependent, R type, alpha 1E subunit | 2.09 |
| VWA5A | von Willebrand factor A domain containing 5A | 2.12 |
| JAM2 | junctional adhesion molecule 2 | 2.13 |
| KLHL6 | kelch-like 6 (*Drosophila*) | 2.14 |
| SLC7A11 | solute carrier family 7, (cationic amino acid transporter, y+ system) member 11 | 2.14 |
| DNAJA1 | DnaJ (Hsp40) homolog, subfamily A, member 1 | 2.16 |
| MPZ | myelin protein zero | 2.16 |
| TCF7 | transcription factor 7 (T-cell specific, HMG-box) | 2.20 |
| ABCB4 | ATP-binding cassette, sub-family B (MDR/TAP), member 4 | 2.23 |
| NEO1 | neogenin homolog 1 (chicken) | 2.23 |
| PPP1R15A | protein phosphatase 1, regulatory (inhibitor) subunit 15A | 2.23 |
| HSPA1L | heat shock 70 kDa protein 1-like | 2.24 |
| KL | klotho | 2.25 |
| SERPINH1 | serpin peptidase inhibitor, clade H (heat shock protein 47), member 1, (collagen binding protein 1) | 2.26 |
| SIRPA | signal-regulatory protein alpha | 2.26 |
| SLC38A6 | solute carrier family 38, member 6 | 2.27 |
| DHRS7 | dehydrogenase/reductase (SDR family) member 7 | 2.29 |
| TSPYL2 | TSPY-like 2 | 2.29 |
| ITPR1 | inositol 1,4,5-triphosphate receptor, type 1 | 2.29 |
| BLVRB | biliverdin reductase B (flavin reductase (NADPH)) | 2.30 |
| KLHL21 | kelch-like 21 (Drosophila) | 2.34 |
| SLC6A9 | solute carrier family 6 (neurotransmitter transporter, glycine), member 9 | 2.35 |
| APLP2 | amyloid beta (A4) precursor-like protein 2 | 2.35 |
| FNIP2 | folliculin interacting protein 2 | 2.36 |
| WDR19 | WD repeat domain 19 | 2.36 |
| MAP1LC3B2 | microtubule-associated protein 1 light chain 3 beta 2 | 2.37 |
| GRB10 | growth factor receptor-bound protein 10 | 2.40 |
| ICK | intestinal cell (MAK-like) kinase | 2.40 |
| LY96 | lymphocyte antigen 96 | 2.40 |
| ESRP1 | epithelial splicing regulatory protein 1 | 2.41 |
| HRG | histidine-rich glycoprotein | 2.42 |
| TTC28 | tetratricopeptide repeat domain 28 | 2.51 |
| PDGFA | platelet-derived growth factor alpha polypeptide | 2.58 |
| ACYP2 | acylphosphatase 2, muscle type | 2.60 |
| SPNS3 | spinster homolog 3 (*Drosophila*) | 2.60 |
| ABHD3 | abhydrolase domain containing 3 | 2.61 |
| AGPAT9 | 1-acylglycerol-3-phosphate O-acyltransferase 9 | 2.63 |
| ASS1 | argininosuccinate synthetase 1 | 2.64 |
| SETD7 | SET domain containing (lysine methyltransferase) 7 | 2.65 |
| ULBP1 | UL16 binding protein 1 | 2.65 |
| SCNN1B | sodium channel, nonvoltage-gated 1, beta | 2.65 |
| HOXB9 | homeobox protein Hox-B9 | 2.73 |
| CYP1A1 | cytochrome P450, family 1, subfamily A, polypeptide 1 | 2.73 |
| EIF4E3 | eukaryotic translation initiation factor 4E family member 3 | 2.74 |
| BVES | blood vessel epicardial substance | 2.81 |
| TRIB3 | tribbles homolog 3 (*Drosophila*) | 2.85 |
| CTSB | cathepsin B | 2.88 |
| INHBE | inhibin, beta E | 2.98 |
| PLEKHC1 | fermitin family homolog 2 (*Drosophila*) | 3.04 |
| RANBP3L | RAN binding protein 3-like | 3.18 |
| UBASH3A | ubiquitin associated and SH3 domain containing, A | 3.23 |
| SQSTM1 | sequestosome 1 | 3.23 |
| UPP1 | uridine phosphorylase 1 | 3.24 |
| NQO1 | NAD(P)H dehydrogenase, quinone 1 | 3.37 |
| GCLM | glutamate-cysteine ligase, modifier subunit | 3.60 |
| TNFRSF9 | tumor necrosis factor receptor superfamily, member 9 | 3.65 |
| SLC48A1 | solute carrier family 48 (heme transporter), member 1 | 3.71 |
| AMBP | alpha-1-microglobulin/bikunin precursor | 3.87 |
| MCOLN3 | mucolipin 3 | 4.05 |
| SLCO2B1 | solute carrier organic anion transporter family, member 2B1 | 4.23 |
| GDF15 | growth differentiation factor 15 | 4.54 |
| CLU | clusterin | 4.76 |
| MT2A | metallothionein 2A | 4.82 |
| HSPA1B | heat shock 70kDa protein 1B | 4.98 |
| SLC30A1 | solute carrier family 30 (zinc transporter), member 1 | 5.13 |
| GPNMB | glycoprotein (transmembrane) nmb | 6.38 |
| CLIC2 | chloride intracellular channel 2 | 6.51 |
| CAB39L | calcium binding protein 39-like | 6.71 |
| MT1X | metallothionein 1X | 9.82 |
| MT1G | metallothionein 1G | 14.38 |
| MT1E | metallothionein 1E | 15.32 |
| MT1F | metallothionein 1F | 27.33 |
| HMOX1 | heme oxygenase (decycling) 1 | 34.93 |

Supplemental Table S2. Biological process allocation of significantly regulated genes by ATO in MEC-1 cells, listed in alphabetical order within each category. See also Figure 2E.

| **Gene name** | **Functional description** | | **Fold change** |
| --- | --- | --- | --- |
| Lipid biosynthetic process, GO:0008610 | | | |
| ACSS2 | | acyl-CoA synthetase short-chain family member 2 | -1.99 |
| COQ2 | | coenzyme Q2 homolog, prenyltransferase (yeast) | -2.51 |
| FDPS | | farnesyl diphosphate synthase (farnesyl pyrophosphate synthetase, dimethylallyltranstransferase, geranyltranstransferase) | -2.46 |
| GNE | | glucosamine (UDP-N-acetyl)-2-epimerase/N-acetylmannosamine kinase | -2.32 |
| HMGCS1 | | 3-hydroxy-3-methylglutaryl-Coenzyme A synthase 1 (soluble) | -2.96 |
| HSD17B8 | | hydroxysteroid (17-beta) dehydrogenase 8 | -2.30 |
| PCYT2 | | phosphate cytidylyltransferase 2, ethanolamine | -2.19 |
| SQLE | | squalene epoxidase | -1.98 |
| Immune response, GO:0006955 | | | |
| CADM1 | | cell adhesion molecule 1 | -2.88 |
| CCR8 | | chemokine (C-C motif) receptor 8 | -4.88 |
| CR5 | | complement component 5 | -2.64 |
| CXCL10 | | chemokine (C-X-C motif) ligand 10 | -6.81 |
| LILRB1 | | leukocyte immunoglobulin-like receptor, subfamily B (with TM and ITIM domains), member 1 | -2.07 |
| PSMB8 | | proteasome (prosome, macropain) subunit, beta type, 8 (large multifunctional peptidase 7) | -2.39 |
| SEMA7A | | semaphorin 7A, GPI membrane anchor | -2.41 |
| **Cell adhesion, GO:0007155** | | | |
| CADM1 | | cell adhesion molecule 1 | -2.88 |
| CCR8 | | chemokine (C-C motif) receptor 8 | -4.88 |
| CNTNAP1 | | contactin associated protein 1 | -2.01 |
| FLRT3 | | fibronectin leucine rich transmembrane protein 3 | -2.66 |
| GNE | | glucosamine (UDP-N-acetyl)-2-epimerase/N-acetylmannosamine kinase | -2.32 |
| RPSA | | 40S ribosomal protein SA | -2.83 |
| **Response to organic substance, GO:0010033** | | | |
| ABCB4 | | ATP-binding cassette, sub-family B (MDR/TAP), member 4 | 2.23 |
| ASAH1 | | N-acylsphingosine amidohydrolase (acid ceramidase) 1 | 2.03 |
| CYP1A1 | | cytochrome P450, family 1, subfamily A, polypeptide 1 | 2.73 |
| DNAJA1 | | DnaJ (Hsp40) homolog, subfamily A, member 1 | 2.16 |
| GRB10 | | growth factor receptor-bound protein 10 | 2.40 |
| HMOX1 | | heme oxygenase (decycling) 1 | 34.93 |
| HSPA1B | | heat shock 70kDa protein 1B | 4.98 |
| HSPA1L | | heat shock 70kDa protein 1-like | 2.24 |
| LY96 | | lymphocyte antigen 96 | 2.40 |
| PDGFA | | platelet-derived growth factor alpha polypeptide | 2.58 |
| PPP1R15A | | protein phosphatase 1, regulatory (inhibitor) subunit 15A | 2.23 |
| SERPINH1 | | serpin peptidase inhibitor, clade H (heat shock protein 47), member 1, (collagen binding protein 1) | 2.26 |
| **Cellular homeostasis, GO: 0019725** | | | |
| APLP2 | | amyloid beta (A4) precursor-like protein 2 | 2.35 |
| CACNA1E | | calcium channel, voltage-dependent, R type, alpha 1E subunit | 2.09 |
| GCLM | | glutamate-cysteine ligase, modifier subunit | 3.60 |
| HMOX1 | | heme oxygenase (decycling) 1 | 34.93 |
| HSPA1L | | heat shock 70kDa protein 1-like | 2.24 |
| ITPR1 | | inositol 1,4,5-triphosphate receptor, type 1 | 2.29 |
| MPZ | | myelin protein zero | 2.16 |
| MT2A | | metallothionein 2A | 4.82 |
| SLC30A1 | | solute carrier family 30 (zinc transporter), member 1 | 5.13 |
| Regulation of apoptotic process, GO:0042981 | | | |
| CLU | | clusterin | 4.76 |
| CTSB | | cathepsin B | 2.88 |
| GCLM | | glutamate-cysteine ligase, modifier subunit | 3.60 |
| HMOX1 | | heme oxygenase (decycling) 1 | 34.93 |
| HSPA1B | | heat shock 70kDa protein 1B | 4.98 |
| MMP9 | | matrix metallopeptidase 9 (gelatinase B, 92kDa gelatinase, 92kDa type IV collagenase) | 2.08 |
| NQO1 | | NAD(P)H dehydrogenase, quinone 1 | 3.37 |
| SQSTM1 | | sequestosome 1 | 3.23 |
| TNFRSF9 | | tumor necrosis factor receptor superfamily, member 9 | 3.65 |
| Response to wounding, GO:0009611 | | | |
| CLU | | clusterin | 4.76 |
| CTSB | | cathepsin B | 2.88 |
| CYP1A1 | | cytochrome P450, family 1, subfamily A, polypeptide 1 | 2.73 |
| HMOX1 | | heme oxygenase (decycling) 1 | 34.93 |
| KL | | klotho | 2.25 |
| LY96 | | lymphocyte antigen 96 | 2.40 |
| PDGFA | | platelet-derived growth factor alpha polypeptide | 2.58 |
| SCNN1B | | sodium channel, nonvoltage-gated 1, beta | 2.65 |
| **Response to unfolded protein, GO:0006986** | | | |
| DNAJA1 | | DnaJ (Hsp40) homolog, subfamily A, member 1 | 2.16 |
| HSPA1B | | heat shock 70kDa protein 1A; heat shock 70kDa protein 1B | 4.98 |
| HSPA1L | | heat shock 70kDa protein 1-like | 2.24 |
| PPP1R15A | | protein phosphatase 1, regulatory (inhibitor) subunit 15A | 2.23 |
| SERPINH1 | | serpin peptidase inhibitor, clade H (heat shock protein 47), member 1, (collagen binding protein 1) | 2.26 |
| **Response to hypoxia, GO:0001666** | | | |
| CYP1A1 | | cytochrome P450, family 1, subfamily A, polypeptide 1 | 2.73 |
| HMOX1 | | heme oxygenase (decycling) 1 | 34.93 |
| ITPR1 | | inositol 1,4,5-triphosphate receptor, type 1 | 2.29 |
| PDGFA | | platelet-derived growth factor alpha polypeptide | 2.58 |
| SCNN1B | | sodium channel, nonvoltage-gated 1, beta | 2.65 |
| **Response to oxidative stress, GO:0006979** | | | |
| CLU | | clusterin | 4.76 |
| GCLM | | glutamate-cysteine ligase, modifier subunit | 3.60 |
| HMOX1 | | heme oxygenase (decycling) 1 | 34.93 |
| NQO1 | | NAD(P)H dehydrogenase, quinone 1 | 3.37 |
| **Porphyrin-containing compound metabolic process, GO:0006778** | | | |
| AMBP | | alpha-1-microglobulin/bikunin precursor | 3.87 |
| CYP1A1 | | cytochrome P450, family 1, subfamily A, polypeptide 1 | 2.73 |
| HMOX1 | | heme oxygenase (decycling) 1 | 34.93 |
| **Response to toxic substance, GO:0009636** | | | |
| CYP1A1 | | cytochrome P450, family 1, subfamily A, polypeptide 1 | 2.73 |
| NQO1 | | NAD(P)H dehydrogenase, quinone 1 | 3.37 |
| SLC7A11 | | solute carrier family 7, (cationic amino acid transporter, y+ system) member 11 | 2.14 |
| **Cellular nitrogen compound catabolic process, GO:0044270** | | | |
| AMBP | | alpha-1-microglobulin/bikunin precursor | 3.87 |
| HMOX1 | | heme oxygenase (decycling) 1 | 34.93 |
| UPP1 | | uridine phosphorylase 1 | 3.24 |
